# Development of a TaqMan-based multiplex real-time PCR for simultaneous detection of porcine epidemic diarrhea virus, *Brachyspira hyodysenteriae*, and *Lawsonia intracellularis*

**DOI:** 10.3389/fvets.2024.1450066

**Published:** 2024-08-14

**Authors:** Jing Ren, Fujun Li, Xue Yu, Yang Li, Meng Li, Yujie Sha, Xiaowen Li

**Affiliations:** ^1^Shandong Engineering Research Center of Swine Health Data and Intelligent Monitoring, Dezhou University, Dezhou, China; ^2^Shandong Provincial Key Laboratory of Biophysics, Institute of Biophysics, Dezhou University, Dezhou, China; ^3^Shandong Engineering Research Center of Pig and Poultry Health Breeding and Important Disease Purification, Shandong New Hope Liuhe Co., Ltd., Qingdao, China

**Keywords:** multiplex real-time PCR, PEDV, *Brachyspira hyodysenteriae*, *Lawsonia intracellularis*, porcine diarrheal diseases

## Abstract

**Introduction:**

PEDV, *Brachyspira hyodysenteriae,* and *Lawsonia intracellularis*, are highly contagious diarrheal pathogens that have caused significant harm to the global swine industry. Co-infections with multiple pathogens are common, making it challenging to identify the actual causative agents depending only on clinical information. It is crucial to develop a reliable method to simultaneously detect and differentiate these pathogens.

**Methods:**

Based on the conserved regions of the M gene of PEDV, NADH oxidase gene of *B. hyodysenteriae*, and the 16S rDNA gene of *L. intracellularis*, specific probes and primers for the multiplex real-time PCR assay were designed. The concentrations of primers and probes were optimized using a matrix method.

**Results:**

The approach demonstrated high specificity and no cross-reactivity with major pathogens related to diarrheal diseases. It showed high sensitivity with a detection limit of 10 copies/μL for *B. hyodysenteriae* and *L. intracellularis*, and 100 copies/μL for PEDV, respectively. It also demonstrated high reproducibility and stability with low coefficients of variation. Results from the multiplex real-time PCR method were in complete agreement with the commercial singleplex real-time PCR kit for detecting PEDV, *B. hyodysenteriae* and *L. intracellularis*. Clinical data revealed single infection rates of 31.46% for PEDV, 58.43% for *B. hyodysenteriae*, and 98.6% for *L. intracellularis*. The co-infection rates were 16.85% for PEDV + *B. hyodysenteriae*, 31.46% for PEDV + *L. intracellularis*, 57.86% for *B. hyodysenteriae* + *L. intracellularis*, and 16.85% for PEDV + *B. hyodysenteriae* + *L. intracellularis*, respectively.

**Discussion:**

The new multiplex real-time PCR method can simultaneously differentiate PEDV, *B. hyodysenteriae* and *L. intracellularis*, making it a valuable diagnostic tool for preventing and controlling infectious diseases, as well as aiding in epidemiological investigations.

## 1 Introduction

Diarrheal disease is a major threat to the global swine industry, causing significant losses in pig production (1, 2). It is caused by various infectious organisms, such as viral and bacterial pathogens. Numerous causative pathogens have been identified in swine, including porcine epidemic diarrhea virus (PEDV), porcine delta coronavirus (PDCoV), transmissible gastroenteritis virus (TGEV), porcine enteric alpha coronavirus (PEAV), porcine rotavirus (PoRV), *Salmonella*, *Escherichia coli*, *Brachyspira hyodysenteriae, Lawsonia intracellularis,* and so on (3). Among these pathogens, PEDV, *B. hyodysenteriae*, and *L. intracellularis* are the most destructive pathogens causing anorexia, diarrhea, dehydration, and vomiting (4–7). With the rapid development of intensive aquaculture, co-infection or secondary infection with these pathogens is prevalent, leading to more severe consequences than single-pathogen infection (4, 8).

Porcine epidemic diarrhea (PED) is a highly contagious diarrheal disease in pigs caused by an enveloped, single-stranded RNA virus belonging to the *Alphacoronavirus* genus in the Coronaviridae famil (9). It is particularly severe in piglets, often leading to 100% mortality (4, 9). PED was first reported in England in 1971, followed by an outbreak in Belgium in 1977, and subsequently identified in China during the 1980s (1, 10, 11). A highly virulent strain emerged in China in December 2010, resulting in over 1 million piglet deaths (11). These strains have since spread worldwide in the swine industry (1, 11).

*B. hyodysenteriae*, a gram-negative anaerobic bacterium, is the classical agent of swine dysentery, a severe mucohaemorrhagic diarrheal disease affecting weanling to finishing pigs (12). This widespread disease can lead to significant mortality rates and decreased feed conversion efficiency, resulting in substantial economic losses for intensive pig production systems globally (12, 13). *L. intracellularis,* a gram-negative obligate intracellular bacterium, is the causative agent of porcine proliferative enteropathy (PPE) (14). PPE is a commonly observed bacterial disease with a high prevalence ranging from 48 to 100% at swine production facilities worldwide (6). Due to the fastidious characteristics of *L. intracellularis*, the obligate anaerobic bacteria are extremely difficult to culture *in vitro* (13, 14).

Rapid and accurate diagnostic methods are essential for effective treatment and prevention programs. However, pigs infected with PEDV, *B. hyodysenteriae*, and *L. intracellularis* show similar symptoms and pathology, making it hard to differentiate them. The high incidence of co-infection with these pathogens further exacerbates the complexities in clinical diagnosis (13). Hence, developing a highly sensitive diagnostic system is necessary to quickly detect and differentiate these causative pathogens to minimize economic losses from diarrheal disease.

Current diagnostic tests for pathogens, such as immunochromatography, antigen detective enzyme-linked immunosorbent assay, conventional PCR, and singleplex real-time PCR, can only detect one pathogen at a time and cannot confirm co-infections (15, 16). Simultaneous detection of multiple pathogens in clinical diagnostics requires multiple reactions, leading to wasted reagents and increased costs. Conversely, multiplex real-time PCR enables the simultaneous detection of multiple pathogens in a single reaction system, making it a widely utilized method in clinical diagnostics (16–18). While numerous multiplex real-time PCR assays have been employed in clinical detection of viral infectious diseases, the simultaneous detection of viral and bacterial pathogens is rarely reported. In this study, we developed a multiple real-time PCR assay using TaqMan probe to simultaneously and accurately detect PEDV, *B. hyodysenteriae*, and *L. intracellularis*. This assay demonstrated high sensitivity and specificity for the target genes, making it a useful tool for rapid pathogen identification.

## 2 Materials and methods

### 2.1 Viruses, bacteria, and clinical samples

Positive samples for various swine pathogens, including PEDV, porcine reproductive and respiratory syndrome virus (PRRSV), porcine circovirus (PCV2, PCV3), African swine fever virus (ASFV), PoRV, PDCoV, *B. hyodysenteriae*, *L. intracellularis*, *Haemophilus parasuis* (HP)*, Streptococcus suis* (SS)*, and Salmonella enteritidis* (SE), confirmed by PCR and DNA sequencing, were stored in our laboratory. A total of 356 clinical samples were collected from pig farms in Shandong and Hebei provinces, including 217 fecal samples and 139 rectal swabs.

### 2.2 Nucleic acid extraction from pathogens

Nucleic acids were extracted from viral and bacterial pathogens, as well as clinical samples, using the NPA-96E Automatic Nucleic Acid Extractors from Bioer Technology Co., Ltd. (Hangzhou, China). The viral nucleic acids were extracted using the VAMNE Virus DNA/RNA Extraction Kit (Nanjing Vazyme Biotech Co.,Ltd.), and bacterial nucleic acids were extracted using the TaKaRa MiniBEST Universal Genomic DNA Extraction Kit (Takara Biomedical Technology (Beijing) Co., Ltd.), following the manufacturer’s guidelines. For RNA viruses, cDNA was synthesized using the TransScript Probe One-Step qRT-PCR SuperMix (Beijing Transgen Biotech Co., Ltd.). The extracted DNA and synthetic cDNA were stored at −80°C until used.

### 2.3 Design of the primers and probes

Primers and probe for PEDV used in the study were previously designed by Ren et al. (4), while those for *B. hyodysenteriae* and *L. intracellularis* were based on at least 30 genome sequences downloaded from NCBI. The primers were designed to target the NADH oxidase gene of *B. hyodysenteriae* and the 16S rDNA gene of *L. intracellularis*. Utilizing Primer Premier 5 software (Premier, Canada), primers and probes were designed based on the most conserved regions. TaqMan probes for PEDV, *B. hyodysenteriae,* and *L. intracellularis* were fluorescently labeled with FAM, VIC, and Cy5 at the 5′ end, respectively, with all quenchers at the 3′ end being BHQ. Sequences of the primers and probes can be found in Table 1 and were synthesized by Sangon Biotech (Shanghai) Co., Ltd.

**Table 1:** Primers and probes designed for the multiplex real-time PCR.

Virus	Primer/probe	Sequence(5′-3′)	Size (bp)	Target gene
PEDV	Forward	CATCTGATTCTGGACAGTTG	226	M
	Reverse	CTATACACCAACACAGGCTC		
	Probe	(FAM)TTTCAGAGCAGGCTGCATAT(BHQ1)		
*L. intracellularis*	Forward	CACCTGGACGATAACTGACACT	110	16 s DNA
	Reverse	TAACTCCCCAGCACCTAGCAC		
	Probe	(CY5) GAGGTGCGAAAGCGTGGGG (BHQ3)		
*B. hyodysenteriae*	Forward	GTAGGAAGAAGAAATCTGACAATGCA	142	NADH oxidase gene
	Reverse	TATGAAGAAGGCAGCAGACGTTTAT		
	Probe	(VIC) GCTTCAGCATGATTGTGT (BHQ1)		

### 2.4 Construction of standard plasmids

The target fragments of PEDV, *B. hyodysenteriae,* and *L. intracellularis* were amplified individually by PCR. The PCR fragments were purified and cloned into the pMD18-T vector (Takara Biomedical Technology (Beijing) Co., Ltd.). The transformed clones were then introduced into the *Escherichia coli* DH5α strain. Positive clones were cultured, and plasmid extraction was done with the TaKaRa MiniBEST Universal Genomic DNA Extraction Kit. The plasmid was confirmed by DNA sequencing and used as the standard positive control. Quantification was done with a UV–visible spectrophotometer, and copy numbers were determined using the following formula (17):


$$Plasmidcopies/\mu L=\frac{\left(6.02\times 10^{23}\right)\times \left(X\;\mathrm{ng}/\mu L\times 10^{-9}\right)}{plasmidlength\left(bp\right)\times 660}$$

A tenfold serial dilution was performed on each plasmid, with concentrations ranging from 1.0 × 10^8^ copies/μL to 1.0 × 10^1^ copies/μL. For the multiplex standard curves, each plasmid was individually diluted to 3.0 × 10^9^ copies/μL and pooled in equal volumes to achieve a final concentration of 1.0 × 10^9^ copies/μL for each plasmid. The combined plasmid solution was then subjected to a tenfold serial dilution, resulting in concentrations ranging from 1.0 × 10^8^ copies/μL to 1.0 × 10^1^ copies/μL, for the establishment of multiplex standard curves.

### 2.5 Optimization of multiplex real-time PCR assay

The concentrations of primers and probes were optimized using a matrix method. Different concentrations of primers (10 μM) ranging from 0.2 to 0.8 μL each, as well as probes (10 μM) ranging from 0.1 to 0.4 μL each, were tested at varying annealing temperatures between 48°C and 57°C to optimize the reaction. Given that PEDV is an enveloped RNA virus, the amplification process was conducted using a one-step reaction, where the entire reaction from cDNA synthesis to real-time PCR amplification was performed in a single well. The main objective was to minimize the Cq value and maximize the fluorescence intensity (RFU). Amplification was carried out on a Bio-Rad CFX96™ Real-time System (Bio-Rad, Hercules, CA, United States), with fluorescence signal being automatically recorded at the end of each cycle. All real-time PCR results were analyzed using CFX Manager™ software.

### 2.6 Sensitivity, specificity, and repeatability test of the multiplex real-time PCR assay

To determine the limit of detection (LOD) for the multiplex real-time PCR method, the aforementioned pooled standard plasmids were diluted in a tenfold serial manner, ranging from 1.0 × 10^8^ copies/μL to 1.0 × 10^−1^ copies/μL in nuclease-free water. These diluted standard plasmids served as templates for the amplification via multiplex real-time PCR, with the reliable LOD being the lowest concentration that achieved a 95% positive detection rate.

To avoid false positives resulting from the presence of other viruses or bacteria in the samples, a specificity test of a multiplex real-time PCR assay was conducted using three RNA viruses (PRRSV, PoRV, and PDCoV), three DNA viruses (PCV2, PCV3, and ASFV), and three bacteria (*H. parasuis, S. suis, and S. enteritidis*). Standard plasmids of PEDV, *B. hyodysenteriae,* and *L. intracellularis* were used as positive controls, with nuclease-free water as the negative control. Three clinical samples from healthy pigs were also tested to confirm specificity.

To test repeatability of the multiplex real-time PCR, pooled standard plasmids with concentrations ranging from 1.0 × 10^6^ copies/μL to 1.0 × 10^4^ copies/μL were used as templates. Each reaction was done in triplicate under identical conditions to assess intra-assay repeatability. Inter-assay repeatability was determined by conducting the assays three times at different time points. The coefficient of variation (CV) of the Cq values was calculated to estimate repeatability, and data analysis was done using Microsoft Excel.

### 2.7 Clinical sample testing

The standard plasmids and ddH_2_O were utilized as positive and negative controls, respectively, in conjunction with optimized reaction conditions for multiplex real-time PCR analysis aimed at detecting the presence of each pathogen. Infection rates were determined by analyzing results from clinical samples.

## 3 Results

### 3.1 Optimization of the reaction conditions for the multiplex real-time PCR

After multiple tests, the optimal reaction conditions for multiplex real-time PCR were determined as follows: 10 μL of 2 × AceQ qPCR Probe Master Mix (Vazyme, Nanjing, China), 1 μL One Step Q Probe Enzyme Mix (Vazyme, Nanjing, China), 0.4 μL each of forward/reverse primers (10 μM), 0.2 μL each of probes (10 μM), 4 μL of template, and ddH_2_O added to a final volume of 20 μL. The reaction program was as follows: 50°C for 5 min, 95°C for 5 min, 40 cycles of 95°C for 10 s, and 52°C for 30 s.

### 3.2 Standard curves of the multiplex real-time PCR

**Figure 1:**
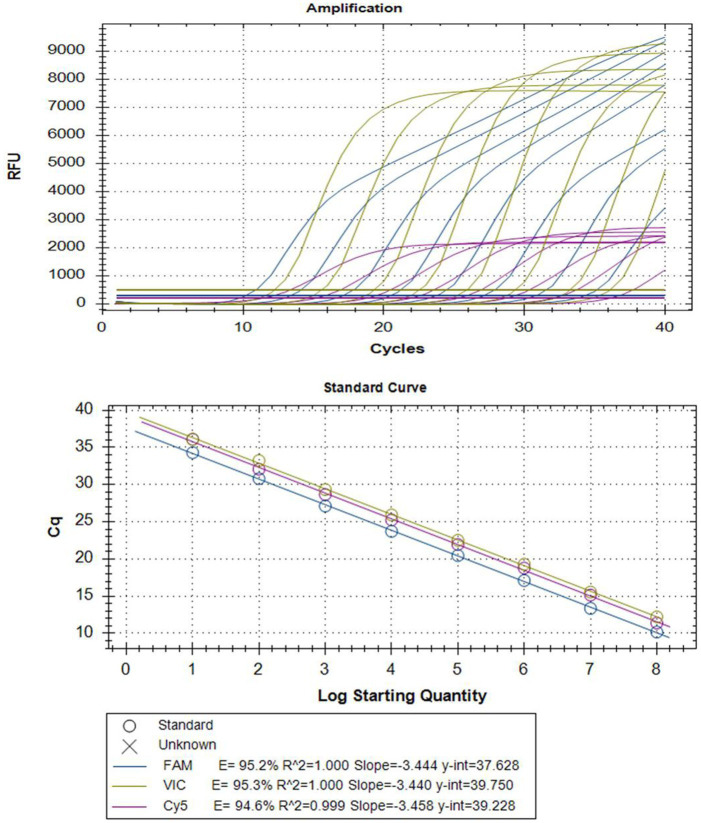
Amplification curves (top) and standard curves (bottom) of optimized multiplex real-time PCR for simultaneous detection of PEDV, *L. intracellularis,* and *B. hyodysenteriae*. The concentrations of each plasmid standard are from 1 × 10^8^ copies/μL to 1 × 10^1^ copies/μL. TaqMan probes for PEDV, *B. hyodysenteriae,* and *L. intracellularis* were fluorescently labeled with FAM, VIC, and Cy5, respectively.

Serial dilutions of mixed plasmid standards were utilized as templates for multiplex real-time PCR amplification with optimized reaction conditions. Standard curves were automatically generated by the fluorescence quantitative PCR instrument, showing high correlation coefficients and amplification efficiency for each pathogen, for details, PEDV (*R*^2^ = 1.000; *E* = 95.2%), *B. hyodysenteriae* (*R*^2^ = 1.000; *E* = 95.3%), and *L. intracellularis* (*R*^2^ = 0.999; *E* = 94.6%) (Figure 1). This result confirms the validity and reliability of the multiplex real-time PCR assay.

### 3.3 The specificity of the multiplex real-time PCR assay

**Figure 2:**
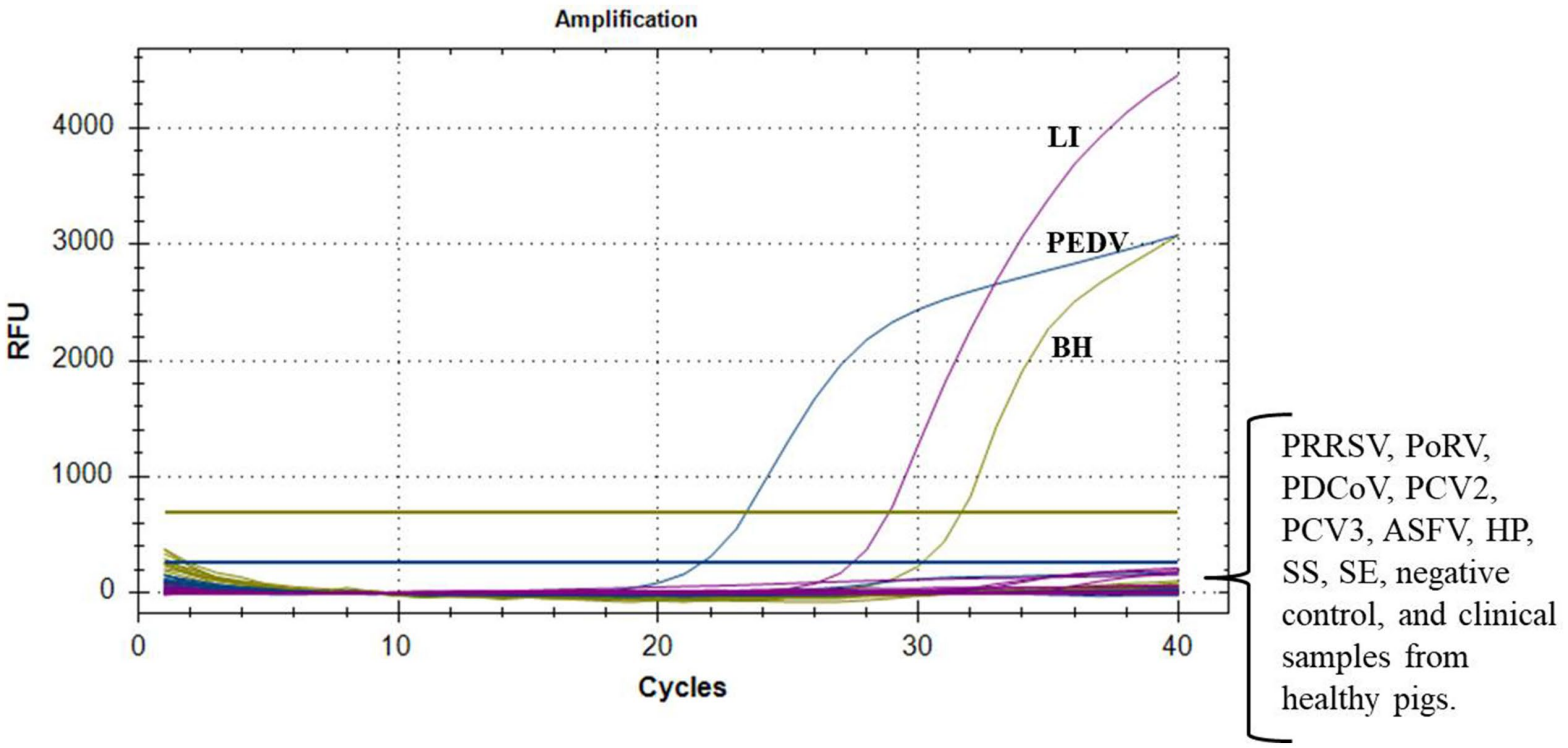
Specificity tests of multiplex real-time PCR. Only PEDV, *L. intracellularis* (LI), and *B. hyodysenteriae* (BH) showed positive fluorescence signals, while other swine pathogens and clinical samples from healthy pigs exhibited no fluorescence signals.

The optimized reaction protocol was utilized for the detection of nucleic acids from a range of porcine pathogens, such as PRRSV, PoRV, PDCoV, PCV2, PCV3, ASFV, *H. parasuis*, *S. suis*, and *S. enteritidis*. As shown in Figure 2, successful detection of all target pathogens was achieved, with no positive signal detected from the aforementioned nine pathogens, the negative control, and three clinical samples from healthy pigs. This finding indicated that the multiplex real-time PCR assay was highly specific, without any cross-reactivity with common pathogens.

### 3.4 The sensitivity of the multiplex real-time PCR assay

**Figure 3:**
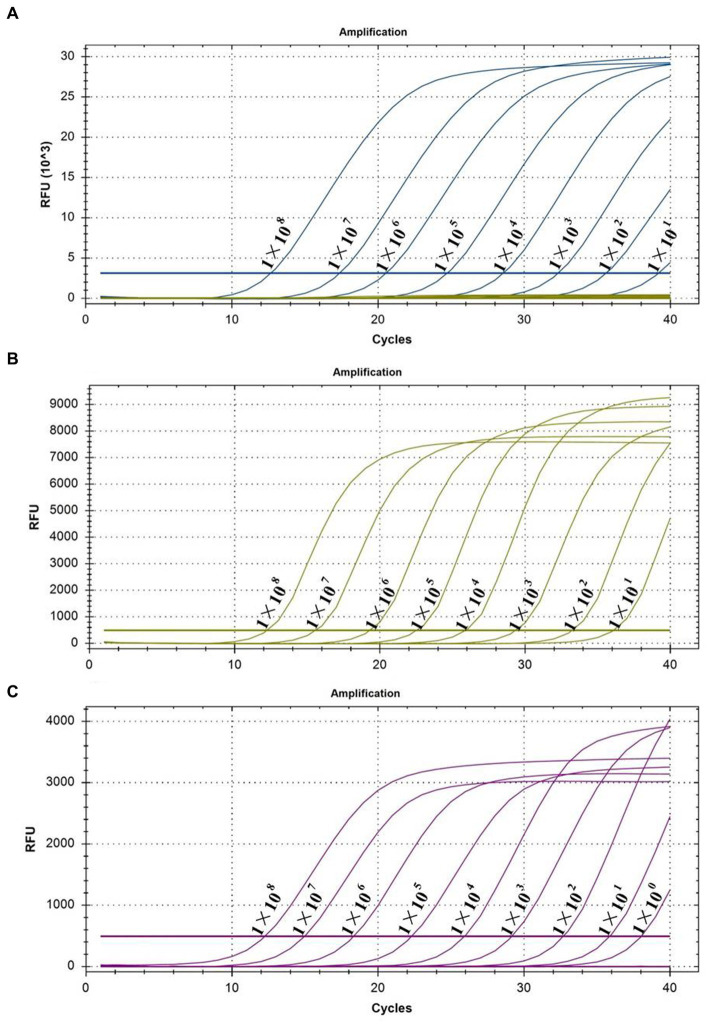
Sensitivity tests of multiplex real-time PCR. **(A)** The test for the sensitivity of PEDV; **(B)** the test for the sensitivity of *B. hyodysenteriae*; and **(C)** the test for the sensitivity of *L. intracellularis*.

The sensitivity of the multiplex real-time PCR assay was tested using different concentrations of pooled standard plasmids, ranging from 1.0 × 10^8^ copies/μL to 1.0 × 10^−1^ copies/μL. Figure 3 shows that the lowest detection limits for PEDV (Figure 3A) and *B. hyodysenteriae* (Figure 3B) were 1.0 × 10^1^ copies/μL, and for *L. intracellularis* was 1.0 × 10^0^ copies/μL (Figure 3C). However, further experiments revealed that the detection rate for *L. intracellularis* and PEDV at those levels was less than 95% of replicates (Supplementary Table S1). Therefore, the reliable detection limit for *B. hyodysenteriae* and *L. intracellularis* is 1.0 × 10^1^ copies/μL, while it is 1.0 × 10^2^ copies/μL for PEDV.

### 3.5 Repeatability of the multiplex real-time PCR assay

The data in Table 2 shows that the variation coefficients of Cq values range from 0.15 to 0.74% in intra-group tests and from 0.12 to 3.5% in inter-group tests. These results suggest the high reproducibility of the multiplex real-time PCR assay developed in this study.

**Table 2:** The repeatability tests of multiplex real-time PCR.

Templates	Concentrations (copies/μL)	Inter-assay		Intra-assay	
		Cq values (mean ± SD)	CV%	Cq values (mean ± SD)	CV%
PEDV	10^4^	29.63 ± 0.47	1.57	30.42 ± 0.18	0.58
	10^5^	25.20 ± 0.18	0.71	25.37 ± 0.07	0.29
	10^6^	19.79 ± 0.69	3.50	20.24 ± 0.03	0.15
*B. hyodysenteriae*	10^4^	31.95 ± 0.21	0.67	32.24 ± 0.13	0.42
	10^5^	27.33 ± 0.06	0.20	27.20 ± 0.09	0.32
	10^6^	22.08 ± 0.31	1.40	22.30 ± 0.04	0.17
*L. intracellularis*	10^4^	30.39 ± 0.19	0.62	30.66 ± 0.11	0.36
	10^5^	25.44 ± 0.08	0.33	25.50 ± 0.19	0.74
	10^6^	20.71 ± 0.03	0.12	20.81 ± 0.09	0.45

### 3.6 Verification of the multiplex real-time PCR assay by commercial singleplex real-time PCR kit

Thirty clinical samples were utilized to conduct a comparative analysis between multiplex real-time PCR and a commercial singleplex real-time PCR kit. Results were consistent between the two methods, indicating that the multiplex real-time PCR assay can replace the commercial singleplex real-time PCR kit for detecting PEDV, *B. hyodysenteriae*, and *L. intracellularis* simultaneously (Supplementary Table S2).

### 3.7 Clinical application of the multiplex real-time PCR

A total of 356 clinical samples were tested using the multiplex real-time PCR assay established in this study. As shown in Table 3, the single infection rates for PEDV, *B. hyodysenteriae*, and *L. intracellularis* were 31.46% (112/356), 58.43% (208/356), and 98.60% (351/356), respectively. Co-infection rates for PEDV + *B. hyodysenteriae*, PEDV + *L. intracellularis*, and *B. hyodysenteriae* + *L. intracellularis* were 16.85% (60/356), 31.46% (112/356), and 57.86% (206/356), respectively. The mixed infection rate for PEDV + *B. hyodysenteriae* + *L. intracellularis* was 16.85% (60/356).

**Table 3:** The detection results of 356 clinical diarrhea samples.

Pathogens	Number of positive samples	Infection rate (%)
PEDV	112	31.46
*B. hyodysenteriae*	208	58.43
*L. intracellularis*	351	98.60
PEDV+ *B. hyodysenteriae*	60	16.85
PEDV+ *L. intracellularis*	112	31.46
*B. hyodysenteriae* + *L. intracellularis*	206	57.86
PEDV+ *B. hyodysenteriae* + *L. intracellularis*	60	16.85

## 4 Discussion

PEDV, *B. hyodysenteriae*, and *L. intracellularis* are highly contagious diarrheal pathogens that have caused significant harm to the global swine industry (4–7). Previously, antibiotics were extensively utilized in animal husbandry to prevent and treat bacterial infections, as well as to promote growth and enhance feed efficiency, resulting in a reduced incidence of bacterial diarrhea. However, the Chinese government implemented a ban on the inclusion of antibiotics in animal feed in 2020. Since the enactment of this regulation, the prevalence of *B. hyodysenteriae* and *L. intracellularis* has been increasing annually. Especially, co-infections with viruses and bacteria are common in some pig herds due to intensive swine production. Distinguishing the specific causative agent based on clinical information alone is difficult due to similarities in symptoms and pathology. Thus, it is essential to develop a reliable method for the differential detection of PEDV, *B. hyodysenteriae*, and *L. intracellularis* in the laboratory and diagnose them accurately in clinical settings.

In this study, three pairs of specific primers and corresponding probes were designed for the conserved regions of the PEDV M gene, *B. hyodysenteriae* NADH oxidase gene, and *L. intracellularis* 16S rDNA gene. Following multiple optimization iterations, a multiplex TaqMan probe-based real-time PCR assay was successfully established for the simultaneous detection of three predominant diarrheal pathogens, namely PEDV, *B. hyodysenteriae,* and *L. intracellularis,* in a single amplification reaction. The method developed in this study is highly sensitive, with a detection limit of 10 copies/μL for *B. hyodysenteriae* and *L. intracellularis*, and 100 copies/μL for PEDV. The multiplex real-time PCR assay also demonstrated good repeatability with coefficients of variation ranging from 0.15 to 0.74% for intra-assays and 0.12–3.5% for inter-assays, which proves the stability and reliability of the results. A comparison was made between a commercial singleplex real-time PCR kit and the multiplex real-time PCR method developed in this study for detecting PEDV, *B. hyodysenteriae*, and *L. intracellularis*, in thirty clinical samples. Results showed complete agreement between the two methods, indicating that the multiplex assay is a viable alternative for simultaneous differentiation of the pathogens.

The multiplex real-time PCR assay developed in this study has been widely applied for the early detection of pathogens in clinical samples due to its rapid, highly sensitive, and specific characteristic. A total of 356 clinical samples from Shandong and Hebei provinces in China were tested using multiplex real-time PCR assay to investigate the prevalence of PEDV, *B. hyodysenteriae*, and *L. intracellularis*. Results showed that *L. intracellularis* and *B. hyodysenteriae* are the main pathogens in diarrheal pigs in both provinces. *L. intracellularis* had the highest infection rate at 98.6%, followed by *B. hyodysenteriae* at 58.43% and PEDV at 31.46%. The prevalence of *B. hyodysenteriae* and *L. intracellularis* infections in Chinese pig herds appears to be higher than previously believed (5, 13). A previous study conducted on 891 fecal samples from 47 farms revealed that 37.3% of the fecal samples and 93.6% of the farms tested positive for *L. intracellularis* (13). Swine dysentery, mainly caused by *B. hyodysenteriae*, was a prevalent disease in China in the 1990s. But with the expansion of large-scale aquaculture in China and the use of antibiotic additives in feed, the incidence of these bacterial diseases has gradually decreased since 2010. Consequently, there is a paucity of research on the current prevalence of these infections. The rise in infection rates of *L. intracellularis* and *B. hyodysenteriae* may be linked to the comprehensive implementation of the ban on the addition of antibiotics in feed in China since 2020.

Co-infections of bacterial and viral pathogens are common in clinical settings and can impact the severity of each other’s infections (19, 20). Our study found that co-infections account for 72.5% (258/356) of samples, suggesting an increasing prevalence of multiple pathogen co-infections associated with expanding large-scale and intensive swine production. Notably, co-infections involving *L. intracellularis* and *B. hyodysenteriae* were found to be prevalent, accounting for 57.86% of cases. Previous study indicated that *L. intracellularis* infection may facilitate the colonization and establishment of *B. hyodysenteriae* in the large intestine, potentially by inducing early changes or impairing the host intestinal immune response (8). This suggests that co-infections of *L. intracellularis* and *B. hyodysenteriae* are common in Chinese pig farms, and need to be addressed for prevention and control.

In conclusion, we have successfully developed a reliable multiplex real-time PCR assay to differentiate PEDV, *L. intracellularis,* and *B. hyodysenteriae.* This assay is highly specific, sensitive, and repeatable, and has shown efficacy in the detection of clinical samples, making it a valuable tool for rapid pathogen identification. Rapid and accurate diagnostics, along with immediate quarantine and treatment, can help prevent and control the spread of infectious diseases.

## Data Availability

The original contributions presented in the study are included in the article/Supplementary material, further inquiries can be directed to the corresponding author.
